# IPP-rich milk protein hydrolysate lowers blood pressure in subjects with stage 1 hypertension, a randomized controlled trial

**DOI:** 10.1186/1475-2891-9-52

**Published:** 2010-11-08

**Authors:** Esther Boelsma, Joris Kloek

**Affiliations:** 1 TNO Quality of Life, Business unit Biosciences, P. O. Box 360, 3700 AJ, Zeist, the Netherlands; 2 DSM Biotechnology Center, P. O. Box 1, 2600 MA, Delft, the Netherlands

## Abstract

**Background:**

Milk derived peptides have been identified as potential antihypertensive agents. The primary objective was to investigate the effectiveness of IPP-rich milk protein hydrolysates (MPH) on reducing blood pressure (BP) as well as to investigate safety parameters and tolerability. The secondary objective was to confirm or falsify ACE inhibition as the mechanism underlying BP reductions by measuring plasma renin activity and angiotensin I and II.

**Methods:**

We conducted a randomized, placebo-controlled, double blind, crossover study including 70 Caucasian subjects with prehypertension or stage 1 hypertension. Study treatments consisted of daily consumption of two capsules MPH1 (each containing 7.5 mg Isoleucine-Proline-Proline; IPP), MPH2 (each containing 6.6 mg Methionine-Alanine-Proline, 2.3 mg Leucine-Proline-Proline, 1.8 mg IPP), or placebo (containing cellulose) for 4 weeks.

**Results:**

In subjects with stage 1 hypertension, MPH1 lowered systolic BP by 3.8 mm Hg (P = 0.0080) and diastolic BP by 2.3 mm Hg (P = 0.0065) compared with placebo. In prehypertensive subjects, the differences in BP between MPH1 and placebo were not significant. MPH2 did not change BP significantly compared with placebo in stage I hypertensive or prehypertensive subjects. Intake of MPHs was well tolerated and safe. No treatment differences in hematology, clinical laboratory parameters or adverse effects were observed. No significant differences between MPHs and placebo were found in plasma renin activity, or angiotensin I and II.

**Conclusions:**

MPH1, containing IPP and no minerals, exerts clinically relevant BP lowering effects in subjects with stage 1 hypertension. It may be included in lifestyle changes aiming to prevent or reduce high BP.

**Trial registration:**

ClinicalTrials.gov NCT00471263

## Background

High blood pressure (BP) is a controllable risk factor in the development of cardiovascular conditions, and therefore any food component with the ability to reduce BP could contribute to the prevention or treatment of cardiovascular diseases [1-3].

Milk-derived peptides have been identified as potential antihypertensive agents. The best-characterized peptides found in fermented or enzymatically treated milk are Isoleucine-Proline-Proline (IPP), and Valine-Proline-Proline (VPP). Over twenty five human studies have been performed linking the consumption of products containing both IPP and VPP with significant reductions in BP [4-6]. Fifteen of the BP studies have been done in Japanese subjects. Ten studies have been performed in Caucasians, that is, in Finnish subjects [7-10], Dutch subjects [11-14], Scottish subjects [15] and American subjects [16].

Effective dosages range from 3.07 mg/d (1.60 mg IPP and 1.47 mg VPP) to 52.5 mg/d (30 mg IPP and 22.5 mg VPP) [10,17]. Recently, casein hydrolysates with different bioactive peptide profiles were developed based on their in vitro ACE inhibitory potential: MPH1 (or tensVida™) and MPH2. The identified peptide contributing to ACE inhibitory effects in MPH1 is IPP, and those in MPH2 are Methionine-Alanine-Proline (MAP), IPP, and Leucine-Proline-Proline (LPP). In the ACE inhibition assays, MPH2 was somewhat more potent than MPH1.

An advantage is that these products contain a negligible amount of minerals contrasting with all other lactotripeptide-based products tested so far [18-23]. Although the dosages of minerals in the sour milk are much lower than those that were effective in lowering BP in intervention trials, and placebo treatments were controlled for mineral content, it is possible that the minerals may have induced additive or synergistic BP effects. To our knowledge, this is the first study performed in Caucasian subjects using products with a negligible amount of minerals.

The primary objective of the study was to investigate the effectiveness of MPHs on reducing BP as well as to investigate safety parameters and tolerability in subjects with prehypertension and stage 1 hypertension. The secondary objective was to obtain information on the mechanism of action underlying the BP effects. The dose chosen is somewhat higher than the lower doses tested for similar products earlier in Japanese subjects, in view of the fact that Caucasian subjects tend to show a smaller blood pressure lowering toward lactotripeptide treatment than Japanese [5,6].

## Methods

### Study participants

Male and female Caucasian subjects were recruited from the pool of volunteers of TNO Quality of Life. After being informed about the study, the subjects gave voluntary written informed consent. Health was assessed by an interview on medical history, physical examination, and routine laboratory tests on blood and urine. To select subjects with prehypertension and stage 1 hypertension, the classification according to JNC-7 was used [24]: subjects with a systolic BP between 140 - 159 mm Hg or a diastolic BP between 90 - 99 mm Hg were assigned to 'stage 1 hypertension', and subjects with a systolic BP between 120 - 139 mm Hg or a diastolic BP between 80 - 89 mm Hg were designated 'prehypertension'. BP was measured on three separate visits. Subjects with a history of medical events or medication that may have influenced the study outcome were excluded from participation. Subjects who were on slimming, medically prescribed, vegan, vegetarian or macrobiotic diet were also excluded as were smokers and subjects using more than 28 units (men) or 21 units (women) of alcohol per week to exclude alcoholics. Pregnant or lactating women were also excluded from participation. Eighty healthy subjects were included in the study.

### Study design

The study was designed as a randomized, placebo-controlled, double blind, crossover study. Study treatments were given to the subjects according to a Latin square design. In view of the very short half-life of lactotripeptides [25], no wash-out period was included between treatments. The study was powered to detect a 3.5 mmHg systolic blood pressure difference between placebo and treatment at an α of 0.05 (two-sided) and a β of 0.8.

### Study protocol

Subjects visited the test facility of TNO Quality of Life at three consecutive test days at the end of each treatment period. At the first two test days, BP measurements were performed two hours after the subjects had their own habitual breakfast and the study substances at home. At the third test day, subjects came in a fasted state for collection of blood and spot-urine samples. No alcohol and sports were allowed the day before the test days, and no dairy products were allowed at breakfast on the test days. During the study period, subjects were instructed not to consume more than one portion of a fermented dairy product per day.

The study was performed according to the ICH Guideline for Good Clinical Practice (ICH topic E6, adopted 01-05-1996 and implemented 17-01-1997) and was approved by the independent Medical Ethics Committee METOPP (Tilburg, the Netherlands).

### Study products

MPH1 and MPH2 were produced through hydrolysis of glycomacropeptide and casein, respectively. Hydrolysis was performed using a proline-specific endoprotease. The nutritional properties, as well as the amounts of bioactive peptides of the study products are presented in table 1. Lactotripeptides were quantified as described in [26].

**Table 1:** Characteristics of study products

	MPH 1	MPH 2	placebo
Protein	570.0	570.0	-
Fat	2.5	2.3	-
Carbohydrates	3.4	13.7	570
Na	3.1	0.9	-
Ca	5.9	1.0	-
K	2.5	34.3	-
Fibre	-	-	570
Ashes (%)	8	5	nd
Total Organic Solids (%)	89	75	nd
IPP	7.5	1.8	-
MAP	-	6.6	-
LPP	-	2.3	-

mg per capsule, unless indicated otherwise

### Study treatments

Study treatments were offered to the volunteers in capsules, to exclude any pre-ingestion interference with food matrices. The treatments consisted of consumption of MPH1 or MPH2 or placebo during 4 weeks. Five hundred mg hydrolysed protein was used per capsule for each of the MPH treatments. The lactotripeptide content per capsule was 7.5 mg IPP for MPH1, and 6.6 mg MAP, 2.3 mg LPP, and 1.8 mg IPP for MPH2. Subjects received 2 capsules per day, and were instructed to consume one capsule directly upon completion of breakfast and one capsule directly upon completion of dinner. Placebo treatment consisted of daily consumption of 2 capsules, each of them filled with 500 mg cellulose (food grade quality).

### Blood pressure measurements

BP was measured between 2.5 and 3.5 h after ingestion of the study substances using automated digital sphygmomanometry (OMRON IC). In brief, subjects rested for at least 15 min at room temperature. The BP cuff was placed around the upper left arm which was supported at the level of the heart while the subject sat in a chair with the feet positioned flat on the floor and with a straight back rested against the chair. The first measurement was taken after three minutes rest. The subject was told not to move or speak. In total four measurements were taken with one minute rest in between. The average of the last three measurements was calculated.

### Safety parameters

Clinical laboratory tests were performed at screening and at the end of treatment and included hematology, serum chemistry (liver enzymes, albumin, bilirubin, urea, creatinin, cholesterol parameters, triacylglycerols, glucose, minerals, blood sedimentation rate, and C-reactive protein). Blood samples were obtained from the antecubital vein of the forearm and collected in tubes containing clot activator for serum and in tubes containing potassium ethylene diamine tetra acid (K_3_EDTA) for plasma (Vacutainer Systems, Becton Dickinson, Plymouth, UK). Blood was centrifuged for 15 min at ca. 2,000 × g at ca. 4°C within 15-30 min after collection, and stored at -20°C until analysis. All biochemical determinations in blood were performed at TNO Quality of Life using Olympus AU400 analytical equipment and reagents. Dipstick urinalysis was performed on the day of collection to assess the presence of protein, glucose, leucocytes, erythrocytes, nitrite, pH, ketones, bilirubin, and urobilinogen. A microscopic inspection of sediment was done if the dipstick test gave values above the normal range for leucocytes, blood or protein.

At the end of treatment, subjects indicated the presence or absence of a number of possible adverse effects in a questionnaire using a 4-point scale from 'not at all' to 'very often'. Symptoms included not feeling well, headache, weakness, fatigue, nausea, vomiting, burping, stomach-ache, bloating, flatulence, constipation, diarrhea, dry mouth, change of taste, cough, and skin complaints.

### Mechanistic parameters

For renin, blood was collected in Vacutainer^® ^tubes containing K3EDTA as anticoagulant and centrifuged immediately (15 minutes at 2,000 × g and ca. 20°C). Plasma was snap frozen in liquid nitrogen and stored at -80°C. For angiotensin I and II, blood was collected in Vacutainer^® ^tubes containing K3EDTA as anticoagulant and protease inhibitor buffer was added at an amount of 50 μL buffer/ml blood. The buffer consisted of (stock 100 mL): 4250 mg EDTA, 8.13 mg enalaprilate, 200 mg neomycin, 0.5 mg o-phenantroline, 2% ethanol, 6 mg renin inhibitor (Sigma, cat.nr. C9415). Samples were centrifuged within 10 minutes after collection (15 minutes at 2,000 × g at 4°C) and plasma was snap frozen in liquid nitrogen and stored at -80°C.

Renin was measured by a standard immunoradiometric assay (Schering SA, CIS bio international, France). Intra- and inter-assay coefficients of variation were 4.1% and 7.6%, respectively. Angiotensin I and II were measured by a standard radioimmunoassay (Peninsula Laboratories, St. Helens, UK) following C-18 Sep-Pak (Waters-Millipore) extraction of the peptide. Intra- and inter-assay coefficients of variation were 4.6% and 7.7%, respectively.

### Statistical analysis

For blood pressure, for every subject and treatment, measurements were first averaged per test day, and subsequently averaged over two successive test days. Statistical analysis of treatment effects on mean DBP and SBP and on mechanistic parameters was performed taking into account the study population, BP class (i.e. prehypertension or stage 1 hypertension), treatment, and interaction between treatment and BP class. Treatment effects were investigated using 2-way ANOVA. Carry over analysis was performed to determine whether a carry over correction was necessary. In case no significant carry over effect was found, the model was simplified by removing this effect. If the analysis of variance indicated an overall treatment effect (P < 0.05), comparisons between treatment means were performed using a 2-sided (paired) Student's t-test. Dichotomized scores in the questionnaire on possible adverse-effects were analyzed using Cochran-Mantel-Haenszel statistics taking into account multiple measurements per subject. In all statistical tests, the null hypothesis was rejected at the 0.05 level of probability (α = 5%). All data are presented as mean ± standard deviation (SD). Statistical analysis of the data was carried out using the SAS statistical software package (SAS/STAT Version 8.2, SAS Institute, Cary, NC).

## Results

### Study participants

Eighty healthy subjects (48 men, 32 women) were included with a mean age of 58 ± 8 y, body mass index (BMI) of 26.2 ± 3.1 kg/m2, systolic/diastolic BP of 136.2 (±12.7)/84.0 (±8.8) mm Hg. Thirty-four subjects had stage 1 hypertension with a mean systolic/diastolic BP of 147.0 (±11.7)/91.5 (±6.1) mm Hg, and 46 subjects had prehypertension with a mean systolic/diastolic BP of 128.1 (±6.1)/78.5 (±6.1) mm Hg. Upon data analysis, placebo BP values were found to be lower than baseline values at the time of screening. Because of this, a number of subjects fell into a lower BP class compared to the onset of the study. As there was no effect of treatment order on BP decrease of either intervention, subjects were classified as having prehypertension or stage 1 hypertension based on placebo values for further analysis of the treatment effects. There was no significant difference between treatments concerning the number of reclassified subjects. This procedure resulted in 26 subjects having stage 1 hypertension, 44 subjects having prehypertension, and 10 subjects having a normal BP (systolic BP < 120 mm Hg and diastolic BP < 80 mm Hg). These normotensives were excluded from further analyses, because the number of subjects was too small for a sound evaluation. Baseline characteristics of the study population (n = 70) are shown in Table 2. Compliance with intake of the study substances, assessed by counting returned capsules, was very good. Also compliance with study procedures with respect to maintenance of habitual diet and physical activity pattern was very good.

**Table 2:** General characteristics of the study population^1^

Parameter	Stage 1 hypertension	Prehypertension
Number of subjects	26	44
Male	17	26
Female	9	18
Age (years)	59.0 ± 7.3	57.5 ± 9.1
Body weight (kg)	82.7 ± 13.8	82.0 ± 12.5
Height (m)	1.8 ± 0.1	1.8 ± 0.1
BMI (kg/m^2^)	26.5 ± 3.4	26.2 ± 3.2
Diastolic BP (mmHg)	89.2 ± 10.1	82.4 ± 7.0
Systolic BP (mmHg)	148.7 ± 11.5	130.3 ± 8.3

^1 ^Data are presented as mean ± SD

### Blood pressure

The BP values after 4 weeks treatment are presented in Table 3. The data indicate that in stage 1 hypertensive subjects, MPH1 induced a significant lowering of systolic BP by 3.8 mm Hg (P = 0.0080) and of diastolic BP by 2.3 mm Hg (P = 0.0065) compared with placebo. In subjects with prehypertension, the differences in systolic BP and diastolic BP between MPH1 and placebo were not significant. MPH2 did not change BP significantly as compared with placebo in either stage I hypertensives or prehypertensives.

**Table 3:** Blood pressure measurements in subjects with stage 1 hypertension and in subjects with prehypertension after daily intake of a placebo, or MPH 1 or MPH 2 for 4 weeks^1^

	Stage 1 hypertension		Prehypertension	
	
	Systolic BP	Diastolic BP	Systolic BP	Diastolic BP
**Placebo**	150.4 ± 8.9	92.0 ± 8.5	128.4 ± 5.1	80.4 ± 4.8
**MPH 1**	146.6 ± 10.8*	89.7 ± 8.7*	130.0 ± 8.1	81.1 ± 6.2
**MPH 2**	149.9 ± 10.0	92.0 ± 7.8	128.9 ± 8.4	81.2 ± 6.2

^1 ^Data are presented as mean ± SD; * denotes significant difference vs. placebo

### Safety parameters

Intake of both MPHs was well tolerated. No significant differences in clinical laboratory parameters and adverse events were observed between placebo and MPHs.

**Figure 1:**
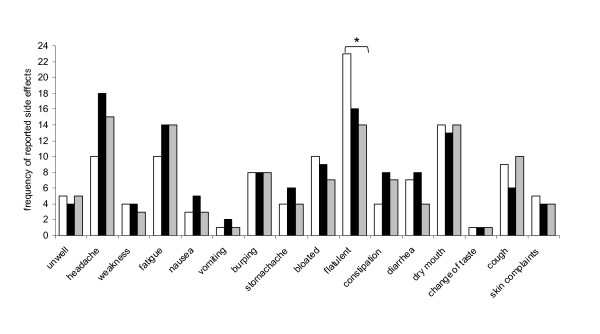
Frequency of reported adverse effects after intake of placebo (white bars) or MPH1 (black bars) or MPH2 (grey bars) for 4 weeks; * significantly different from placebo, P < 0.05.

Thirty seven subjects reported a adverse effect after intake of the placebo (in total 118 occurrences of adverse effects), 43 subjects reported a adverse effect after intake of MPH1 (in total 126 occurrences of adverse effects), and 37 subjects reported a adverse effect after consumption of MPH2 (in total 114 occurrences of adverse effects). Most reported symptoms occurred 'hardly ever' or 'sometimes'. In single occasions, a symptom occurred 'often' or 'very often', but no clear differences were observed between treatments. Adverse effects most often reported were flatulence, headache, dry mouth, and fatigue. Only flatulence was significantly more often reported after placebo treatment (23×) compared with MPH1 (16×; P = 0.0196) and MPH2 (14×; P = 0.0339) (Figure 1). Since the frequency and seriousness of the reported adverse effects was low, the above mentioned differences between MPHs and placebo were not considered clinically relevant.

### Mechanistic parameters

**Figure 2:**
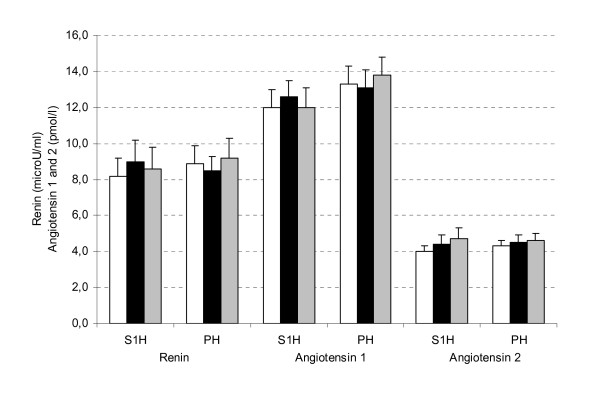
**Plasma renin activity and angiotensin 1 and 2 concentrations (mean ± SEM) in stage 1 hypertensive subjects (S1H, n = 26) and prehypertensive subjects (PH, n = 44) and after intake of placebo (white bars) or MPH1 (black bars) or MPH2 (grey bars) for 4 weeks**.

No significant differences between MPHs and placebo treatment were found in plasma renin activity or concentrations of angiotensin I and II (Figure 2). In all subjects, intake of the placebo, MPH1, and MPH2 for 4 weeks resulted in similar mean renin activities. Mean angiotensin I and II concentrations after intake of placebo were comparable with those after intake of MPH1 and MPH2.

## Discussion

In subjects with stage 1 hypertension, daily intake of MPH1, delivering 15 mg IPP, for 4 weeks lowered systolic BP by 3.8 mm Hg and diastolic BP by 2.3 mm Hg compared with placebo. Daily intake of MPH2 containing 13.2 mg MAP, 4.6 mg LPP, and 3.7 mg IPP did not affect BP in these subjects. Since MPH1 contains primarily IPP while MPH2 contains mainly MAP and LPP, this differential effect despite equal protein dose of the two treatments suggests superiority of IPP over the other peptides for BP lowering, or even functional antagonism of MAP and LPP against IPP (i.e. MAP and LPP having an opposite effect compared to IPP in vivo). Alternatively, MAP and LPP may be less bioavailable than IPP. The bioavailability of IPP has been demonstrated in humans [25], but bioavailability data for MAP and LPP are lacking.

In our study MPHs were given for a period of 4 weeks. A further lowering of BP might have been achieved in a longer treatment periods as has been shown in studies on comparable products [27-30].

In subjects with prehypertension, daily intake of both MPH1 and MPH2 did not affect BP. Also in other studies, lactotripeptides, in particular VPP and IPP, appear more effective in reducing BP of subjects with a higher starting BP (Sano et al, 2005; Aihara et al, 2005). Indeed, in none of the trials with normotensives were any statistically significant BP changes found [31-34]. This is in line with findings in studies using pharmaceutical interventions [35].

In many papers, BP reduction through lactotripeptides has been suggested to be due to inhibition of the renin-angiotensin system, which plays an important role in regulating arterial pressure. Renin converts angiotensinogen to the biologically inactive angiotensin I, which in turn undergoes proteolytic cleavage by ACE to the vasoconstrictor angiotensin II [36]. Inhibition of ACE leads to an increase of the angiotensin I/angiotensin II ratio and a subsequent compensatory increase in renin activity. We could not demonstrate any effects of either MPH on renin activity, angiotensin I and II. Although local ACE inhibitory effects near the vascular wall may be overlooked by measuring angiotensin in blood samples from the systemic circulation, these findings do not support an angiotensin-mediated effect of ACE inhibition in vivo. This may explain why the in vitro ACE inhibitory potency of MPH1 and MPH2 did not predict their BP lowering activity. Indeed, based on their IC50 values, MPH2 was expected to lower BP more than MPH1, but the contrary was found in this study.

An alternative way in which ACE inhibition could be involved in the observed BP effects would be through an increased availability of the endogenous vasodilator bradykinin [37]. Alternative mechanisms, such as such as opioid-like activities, inhibition of the release of the vasoactive substances such as the vasoconstrictor endothelin-1, eicosanoids and nitric oxide have been proposed to underlie the blood pressure lowering effect of lactotripeptides (see [38] for a review). Further work is needed to better characterize these mechanisms.

Since lactotripeptides are frequently taken in a dairy drink or a similar vehicle, it is often unclear whether the blood pressure lowering effects are solely due to the lactotripeptides, or whether minerals in these drinks contribute to the blood pressure lowering effects as well. In the current study, the lactotripeptides have been administered in capsules rather than in dairy drinks. Also, MPH1 contains very few minerals (1.1 mg K^+ ^+ Ca^2+ ^per mg IPP) compared to other products that have been administered in capsules in other studies (2.3 - 3.6 mg K^+ ^+ Ca^2+ ^per mg IPP) [28,31,33,39]. Therefore, it is very unlikely that minerals have contributed to the observed blood pressure lowering effect.

The doses of MPH used in this study did not exert any significant effects on blood and urine parameters, adverse events, and other adverse effects, and were thus considered safe. Previous studies confirmed that even high daily dosages of VPP and IPP were safe [10,31,33,34,39].

The BP reductions found for MPH1 are in the order of magnitude of those that can be achieved by lifestyle modifications, such as weight loss in overweight subjects [40], regular aerobic exercise [41,42], adjustment of dietary habits [43], and moderation of alcohol consumption [44]. Cook and co-workers [45] and Whelton and co-workers [41] investigated the impact of small reductions in the population distribution of diastolic BP, such as those found in our study and those potentially achieved by lifestyle modification, on incidence of cardiovascular heart disease and stroke. Cook estimated that a 2 mm Hg reduction in the population average of diastolic BP for white US subjects aged 35-64 y would result in a 17% decrease in the prevalence of hypertension, a 15% reduction in the risk of stroke, and a 6% reduction in the risk of coronary heart disease.

## Conclusions

MPH1, an IPP-rich milk protein hydrolysate, is safe and exerts relevant BP lowering effects in subjects with stage 1 hypertension. It may be included in lifestyle changes aiming to prevent or reduce high BP.

## Competing interests

E.B. did not have a conflict of interest. J.K. is employed as a senior scientist at the Department of Nutrition and Health at DSM Biotechnology Center, the Netherlands.

The study was financially supported by DSM Food Specialties, Delft, the Netherlands, the manufacturer of the study substances.

## Authors' contributions

EB and JK were responsible for design of the study; EB was responsible for conduct of the study and data interpretation; EB and JK wrote the manuscript; Both JK and EB read and approved the final version.
